# Draft genome sequences of multi-drug-resistant *Escherichia coli* strains isolated from ulcerative colitis patients

**DOI:** 10.1128/mra.00719-24

**Published:** 2024-11-14

**Authors:** Asha Yadav, Pratik Balwant Shinde, Mahesh S. Dhar, Kalaiarasan Ponnusamy, Robin Marwal, RadhaKrishnan VS, Saurabh Kedia, Vineet Ahuja, Krishna Kant Sharma

**Affiliations:** 1 Laboratory of Enzymology and Gut Microbiology, Department of Microbiology, Maharshi Dayanand University, Rohtak, Haryana, India; 2 Sunny Corporation Pvt Ltd, New Delhi, India; 3 Department of Biotechnology, National Centre for Disease Control, Delhi, India; 4 Department of Gastroenterology and Human Nutrition, All India Institute of Medical Sciences, New Delhi, India; University of Maryland School of Medicine, Baltimore, Maryland, USA

**Keywords:** *Escherichia coli*, whole-genome sequence, ulcerative colitis, antibiotic resistance

## Abstract

The whole-genome sequences of four multi-drug-resistant *Escherichia coli* strains, namely, DAK02, DAK03, DAK05, and DAK10, are reported here. The *E. coli* strains were isolated from fecal samples of ulcerative colitis patients from Northern India. The size of the draft genome sequence ranged from 4,809 to 5,000 kb.

## ANNOUNCEMENT

*Escherichia coli* is a Gram-negative rod-shaped bacterium of the family *Enterobacteriaceae*. It is a versatile bacterium that plays a dual role as some strains are beneficial that aid in digestion and vitamin K production, whereas others are highly pathogenic, causing diarrhea, abdominal cramps, urinary tract infections, and meningitis (1, 2).

In the current study, four *E. coli* strains, DAK02, DAK03, DAK05, and DAK10, were isolated from the feces of ulcerative colitis (UC) patients from Northern India region. The *E. coli* strains were isolated on Sakazakii Deoxycholate–Hydrogen sulfide–Lactose Agar (DHL) (HiMedia, GM1619) using 1 mg feces diluted in phosphate-buffered saline (PBS). The dilution (0.1 mL of the 10^−4^) was spread on DHL agar plates and incubated at 37°C/16 hours under aerobic conditions. The characteristic green metallic sheen colonies were observed on eosin methylene blue (EMB) medium. The microscopic examination at 1,000 x magnification shows Gram-negative rods. The biochemical characterization is suggestive of nitrate-positive, oxidase-negative, indole-positive, citrate-negative, catalase-positive, and presence of mixed-acid fermentation (3). The antimicrobial susceptibility profiling depicted that isolates were resistant to multiple drugs of different classes, *viz*., β-lactams, cephems, fluoroquinolones, nitrofurans, trimethoprim, sulfonamides, and tetracyclines (Table S1). The antimicrobial susceptibility testing was performed according to Clinical and Laboratory Standards Institute (CLSI) guidelines by Kirby–Bauer disk diffusion method using HiMedia antibiotic disks (4, 5). Further, the isolates were serotyped at “National *Salmonella* and *Escherichia* Centre,” Central Research Institute (CRI), Kasauli, Himachal Pradesh, India (Table 1).

**TABLE 1:** Summary of the whole-genome data of different *Escherichia coli* strains isolated from ulcerative colitis patients^*a*^

	*Escherichia coli* strains			
	DAK02	DAK03	DAK05	DAK10
No. of Illumina reads (after QC)	1527733	990225	5628026	2756568
Size of reads	130–133	130–133	130–133	130–133
Genome coverage	61	38	221	117
Genome GC content (%)	50.76	50.7	50.76	50.81
Genome size (bp)	4886397	5000688	4886958	4809628
N50 size (bp)	100770	115488	102361	94420
L50 size (bp)	15	14	15	17
No. of contigs	142	126	145	139
No. of predicted genes	4,774	4,895	4,774	4,713
No. of predicted proteins	4,472	4,576	4,473	4,446
No. of RNA genes	67	67	67	66
No. of ARGs	70	58	70	60
BLAST	*E. coli*	*E. coli*	*E. coli*	*E. coli*
SILVA	*E. coli*	*E. coli*	*E. coli*	*E. coli*
TYGS	*E. coli*	*E. coli*	*E. coli*	*E. coli*
*E. coli* serotype	O84	O101	O11	O76
BioSample number	SAMN35982364	SAMN35982365	SAMN35982363	SAMN35982362
SRA accession number	SRR29063301	SRR29063300	SRR29063302	SRR29063303
GenBank	JAUIQR000000000	JAUIQS010000000	JAUIQQ010000000	JAUIQP010000000

^*a*^ ARG, Antibiotic resistant gene; QC, Quality control; BLAST, Basic Local Alignment Search Tool; TYGS, Type (Strain) Genome Server; SRA, Sequence Read Archive.

Each isolate was grown in the nutrient broth at 37°C/16 hours and processed for genomic DNA extraction according to the phenol–chloroform method. The extracted DNA quality was checked on 0.8% agarose gel and quantified. DNA libraries were prepared using the Illumina DNA prep kit. The paired-end whole-genome sequencing was carried out on an Illumina MiSeq platform. The raw reads were quality-checked using FastQC v.0.12.0 (6) and quality-controlled using Trimmomatic v0.32 (7) for the removal of low-quality reads, adapters, and primer sequences (ILLUMINACLIP: NexteraPE-PE.fa:2:30:10 LEADING:3 TRAILING: 3 SLIDINGWINDOW:4:15 HEADCROP:15 CROP: 133 MINLEN:130). The genome sequences of the isolates were assembled using SPAdes v3.15.5 (8) “-isolate” command, and assembly quality was assessed by CheckM v1.2.2 (9). The taxonomy analysis of each isolate was accomplished using 16 s rRNA gene aligned using BLAST +v2.14.1 (10), against the SILVA v132 database (11), and Type (Strain) Genome Server (TYGS, https://tygs.dsmz.de). The genome assemblies uploaded on GenBank were annotated using PGAP v6.7 (12). In addition to that, antimicrobial resistance (AMR) genes were identified in the genome of each isolate by passing contigs through the CARD-RGI v6.0.2 tool (13). All the software were used with default parameters unless otherwise specified.

The information about whole-genome sequences and genome characteristics for each *E. coli* strain is detailed in Table 1. The reported genome sequences of multi-drug-resistant *E. coli* strains will improve our knowledge to demystify *E. coli* dynamics in the UC gut. It allows bacterial characterization in terms of molecular epidemiology, antibiotic resistance, and virulence factors. The current information provides a platform to illustrate disease-causing mechanisms and their role in UC pathogenesis.

## Data Availability

This Whole-Genome Shotgun sequences has been deposited at the NCBI under BioProject ID: PRJNA987404.
